# Engineering Ferroptosis Inhibitors as Inhalable Nanomedicines for the Highly Efficient Treatment of Idiopathic Pulmonary Fibrosis

**DOI:** 10.3390/bioengineering10060727

**Published:** 2023-06-17

**Authors:** Mengqin Guo, Tingting Peng, Chuanbin Wu, Xin Pan, Zhengwei Huang

**Affiliations:** 1 College of Pharmacy, Jinan University, Guangzhou 511436, China; guomengqin@stu2021.jnu.edu.cn (M.G.); chuanbinwu@jnu.edu.cn (C.W.); 2 School of Pharmaceutical Sciences, Sun Yat-sen University, Guangzhou 510006, China; panxin2@mail.sysu.edu.cn

**Keywords:** ferroptosis inhibitor, idiopathic pulmonary fibrosis, inhalable, nanomedicine, micelles, liposomes, nanocrystals

## Abstract

Idiopathic pulmonary fibrosis (IPF) refers to chronic progressive fibrotic interstitial pneumonia. It is called a “tumor-like disease” and cannot be cured using existing clinical drugs. Therefore, new treatment options are urgently needed. Studies have proven that ferroptosis is closely related to the development of IPF, and ferroptosis inhibitors can slow down the occurrence of IPF by chelating iron or reducing lipid peroxidation. For example, the ferroptosis inhibitor deferoxamine (DFO) was used to treat a mouse model of pulmonary fibrosis, and DFO successfully reversed the IPF phenotype and increased the survival rate of mice from 50% to 90%. Given this, we perceive that the treatment of IPF by delivering ferroptosis inhibitors is a promising option. However, the delivery of ferroptosis inhibitors faces two bottlenecks: low solubility and targeting. For one thing, we consider preparing ferroptosis inhibitors into nanomedicines to improve solubility. For another thing, we propose to deliver nanomedicines through pulmonary drug-delivery system (PDDS) to improve targeting. Compared with oral or injection administration, PDDS can achieve better delivery and accumulation in the lung, while reducing the systemic exposure of the drug, and is an efficient and safe drug-delivery method. In this paper, three possible nanomedicines for PDDS and the preparation methods thereof are proposed to deliver ferroptosis inhibitors for the treatment of IPF. Proper administration devices and challenges in future applications are also discussed. In general, this perspective proposes a promising strategy for the treatment of IPF based on inhalable nanomedicines carrying ferroptosis inhibitors, which can inspire new ideas in the field of drug development and therapy of IPF.

## 1. Introduction

Idiopathic pulmonary fibrosis (IPF) is a specific form of chronic progressive fibrotic interstitial pneumonia having complicated causes [1].Its pathology is characterized by the replacement of healthy tissue by an altered extracellular matrix and the destruction of alveolar structure, resulting in reduced lung capacity, disruption of gas exchange, and ultimately respiratory failure and death [2]. The estimated incidence of IPF (per 10,000 people) was 0.57 to 4.51 in Asia-Pacific countries, 0.33 to 2.51 in Europe, and 2.40 to 2.98 in North America [3]. Current clinical treatments include drug palliative care and lung transplantation, with a median survival rate of 50% at three years and 20% at five years after diagnosis, with significant morbidity and poor survival [3]. The average annual cost for an IPF patient in the United States was approximately $20,000, three times the U.S. national average per capita health expenditure. In a study conducted in Canada, the cost of IPF treatment was more than three times higher than the per capita national health expenditure [4]. China also initiated a prospective, multicenter clinical study of IPF in 2020, which showed significant use of manpower and material resources [5]. This evidence demonstrated that IPF is becoming a growing socioeconomic burden on the global public [4].

**Figure 1:**
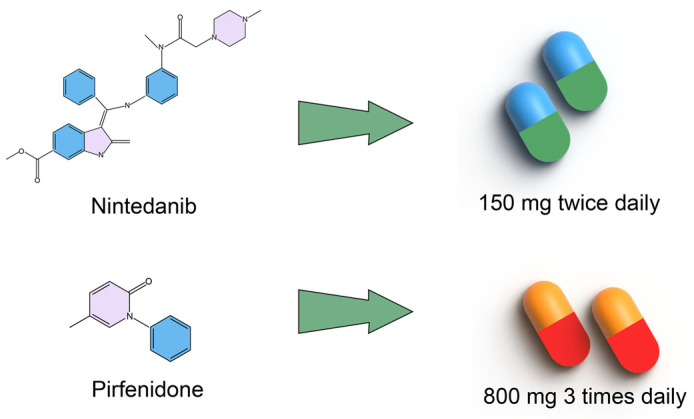
Drug therapy for IPF in clinic.

Currently, drug therapy is widely used to manage IPF. Nonetheless, drug therapy cannot completely cure IPF, and the goal of patient care is now to improve patient prognosis by slowing disease progression, prolonging life, and improving quality of life. Two drugs, nintedanib and pirfenidone, are approved for the treatment of IPF [6]. Nintedanib is a tyrosine kinase inhibitor with affinity for three targets: vascular endothelial growth factor (VEGF), fibroblast growth factor (FGF), and platelet-derived growth factor (PDGF) [7]. Based on the results of the TOMORROW and INPULSIS^®^ trials, nintedanib 150 mg twice daily (Figure 1) significantly reduced the annual rate of decline in forced vital capacity (FVC) and reduced the rate of exacerbations compared with a placebo [8]. However, adverse events reported in >10% of nintedanib-treated patients included diarrhea, nausea, abdominal pain, vomiting, elevated liver enzymes, etc. Pirfenidone is a transforming growth factor beta (TGF-β) inhibitor with important functions in the regulation of pro-fibrotic cascades, fibroblast proliferation, and collagen synthesis [7]. Based on the results of the ASCEND and CAPACITY studies, pirfenidone 800 mg three times daily (Figure 1) significantly reduced the decline in FVC compared to a placebo [8]. However, the use of pirfenidone in the treatment of IPF also has adverse reactions of varying degrees, including nausea, rashes, abdominal pain, upper respiratory tract infections, diarrhea, etc. Although two treatments have been approved for IPF on the basis of reduced lung-function decline, neither has a significant advantage in terms of mortality outcomes [9]. Of note, nintedanib and pirfenidone are both administered orally [10]. Oral administration is the preferred mode for most drugs because of its convenience and high patient compliance. However, oral administration involves crossing a strict physiological barrier in the gastrointestinal tract, which significantly reduces the bioavailability of the drug at the site of the disease (i.e., the lung), and therefore often requires higher doses for administration, which not only increases the drug requirement but also causes different degrees of side effects [11]. Based on the above statements, a better drug and a more suitable delivery method are urgently needed for the treatment of IPF.

## 2. Ferroptosis Inhibition Is a Candidate Therapy for IPF

To date, the detailed pathogenesis of IPF has not been clearly elucidated, and the two major existing theories are proposed based on the stimulus of inflammatory factor and cytokines.

Theory 1: Inflammatory stimuli can cause the spread of immunopathology, tissue destruction, and wound-healing responses [12]. Based on this theory, IPF is considered to result from chronic inflammatory stimulation via diverse pathogenic factors, which eventually lead to persistent lung injury and pulmonary fibrosis. During the early stages, inflammatory cells are involved in normal wound healing. If the inflammatory response persists, it will promote local fibroblast migration and hyperproliferation, accelerate the differentiation of fibroblasts into activated myofibroblasts, and further cause lung-tissue fibrosis. Specifically, this process involves two pro-fibrotic factors, IL-4 and IL-13 [12]. As a direct mechanism, IL-4 receptors are present on lung fibroblasts, and increased IL-4 signaling enhances extracellular matrix protein and collagen deposition [13]. Via an indirect mechanism, IL-4 can promote the ability of macrophages (AA-Mac) to activate alternatively. AA-Macs can produce TGF-β, PDGF, and regulate polyamine and proline biosynthesis, cell growth, and collagen formation through arginase upregulation [14]. In addition, IL-4 can induce many type-two cytokines to the inflammatory axis (IL-5, IL-9, IL-13 and IL-21) by promoting the differentiation of T cells into Th2 cells, which in turn exacerbates fibrosis [12]. IL-13 is a key fibrotic cytokine that acts independently of TGF-β. It triggers fibroblast differentiation into α-smooth muscle actin (α-SMA)-expressing myofibroblasts and PDGF-producing cells with significant mitogenic properties that promote collagen production [15]. In addition to the direct effect of inflammatory factors, the occurrence of inflammation also prompts macrophages to mediate lung-tissue damage. During injury repair, M2-type macrophages thicken lung mucus, cause abnormal deposition of an extracellular matrix in the lung interstitium, and exacerbate IPF [16]. This theory is to some degree prevailing currently.

Theory 2: Epithelial-cell-derived TGF-β triggers a fibrotic cascade of increased interstitial collagen and fibroblast proliferation in the absence of inflammation [12]. This theory holds that cytokines such as TGF-β can mediate fibroproliferation and further activate inflammatory cells, thereby producing a series of chain reactions and a cascade of negative effects. TGF-β plays a key role in the inflammation, injury, and repair of IPF, and its signal-transduction pathway within the Smad family plays a significant regulatory role [17]. This theory is at a relatively complementary status.

Notably, with the deepening of research on lung diseases, scientists think that ferroptosis may play a critical role in the occurrence and development of IPF. Ferroptosis was firstly introduced by Dixon et al. in 2012, and it was defined as an iron-dependent form of non-apoptotic cell death manifested by the accumulation of intracellular iron, depletion of glutathione, inactivation of glutathione peroxidase 4 (GPX4), and promotion of lipid peroxidation [18]. At present, research on ferroptosis is still in its infancy, and it has been confirmed that ferroptosis is regulated by multiple genes, mainly involving gene changes in iron homeostasis, lipid-peroxidation metabolism, and amino acid metabolism. In the past decade since ferroptosis was proposed, a large number of clinical and basic studies have confirmed that ferroptosis is involved in the occurrence of various diseases, such as acute traumatic injury, neurodegenerative diseases, stroke, and cancer. However, many studies in recent years have shown that the incidence of various lung-related diseases such as lung cancer and acute lung injury is closely related to ferroptosis. Fu et al. [19] utilized dihydroartemisinin to sensitize A549 ferroptosis to accelerate tumor elimination in vitro and in vivo. Liu et al. [20] found that the ferroptosis inhibitor ferrostatin-1 (Fer-1) effectively alleviates lipopolysaccharide (LPS)-induced acute lung injury and inflammatory response. Likewise, IPF was also found to be associated with the ferroptosis pathway. It was documented that in the bleomycin-induced IPF model in mice, a large number of iron deposition and typical ferroptosis-like mitochondrial ultrastructural changes appeared in alveolar type II (ATII) cells, indicating that ATII cell ferroptosis and IPF occurrence and development are closely related [21].

Therefore, inhibition of ferroptosis will possibly emerge as a promising approach to the treatment of IPF. Encouragingly, increasing studies have demonstrated the inhibitory efficacy of ferroptosis inactivation on IPF, as detailed below. 

Cheng et al. [21] found abundant iron deposits in the lung tissue of patients with IPF. They also found that the ferroptosis-inhibitor deferoxamine (DFO) completely prevented ATII cell damage and the profibrotic effects of bleomycin (BLM) by reducing iron deposition. According to the survival-rate curve, the survival rate of mice in the BLM-induced IPF model was 50% at day 21 and increased to 90% with the addition of DFO, indicating that the progression of IPF disease could be significantly reversed and that the survival rate of model mice could be improved by inhibiting ferroptosis in ATII cells. Gong et al. [22] used an HFL1 cell line treated with transforming growth factor-β1 to study the relationship between ferroptosis and IPF. At the in vitro level, the expression levels of α-SMA and collagen I significantly increased after TGF-β1 treatment for 24 h, and further increased after TGF-β1+erastin treatment. At the same time, the ferroptosis indicators of the TGF-β1+erastin treatment group also changed correspondingly, such as the increase of ROS and malondialdehyde (MDA) levels, and the decrease of GPX4 mRNA and protein levels. After pretreatment with TGF-β1+erastin, the addition of Fer-1 was found to restore the TGF-β1- and erastin-induced changes. In addition, the survival rate of cells treated with TGF-β1+erastin was the lowest, which was significantly different from other control groups, suggesting that induction of ferroptosis may accelerate the death of HFL1 cells to aggravate IPF. Collectively, the results indicated that the ferroptosis-inducer erastin promoted fibroblast-to-myofibroblast differentiation by increasing lipid peroxidation and inhibiting the expression of GPX4. Fer-1 may inhibit ferroptosis by inhibiting lipid peroxidation and enhancing GPX4 expression and further inhibit IPF. Yuan et al. [23] found that dihydroquercetin (DHQ) attenuated silica-induced pulmonary fibrosis by inhibiting the ferroptosis-signaling pathway. DHQ ameliorated pulmonary fibrosis by reducing the accumulation of iron and lipid peroxidation products, significantly inhibiting ferroptosis in activated human bronchial epithelial (HBE) cells. From the in vivo results, DHQ treatment significantly attenuated the SiO_2_-induced weight loss. Compared with the control group, the body weight of mice in the model group was sharply reduced by 10 g. When DHQ was treated, the body weight of mice was reduced by only about 5 g compared with the control group. Also, the expression of IPF biomarkers such as α-SMA, collagen I, and fibronectin was significantly reduced in the DHQ-treated mice. These results further demonstrated that by inhibiting ferroptosis, DHQ could greatly reduce the extent of SiO_2_-induced inflammation and fibrosis in lung tissue. This evidence suggests that ferroptosis inhibitors might provide potential targets for the treatment of IPF. 

## 3. Inhalable Nanomedicine Is Suitable for Ferroptosis Inhibitors Delivery

**Figure 2:**
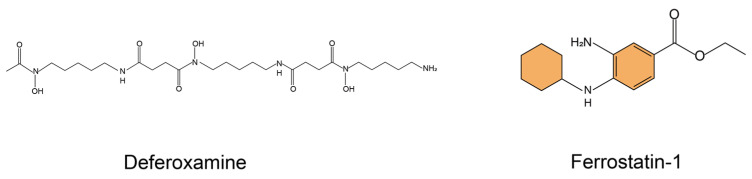
Molecular structural formula of classical ferroptosis inhibitors.

Ferroptosis inhibitors have emerged as a promising remedy to slow down the progression of IPF. Currently, classical ferroptosis inhibitors usually include the iron chelator DFO, the aromatic amine antioxidants Fer-1 [24] and liproxstatin-1 (Lip-1) [25], α-tocopherol analogs [26], and some polyphenolic natural compounds such as baicalein [27] and curcumin (CN) [28]. Among them, the commonly-used ferroptosis inhibitors are DFO and Fer-1(Figure 2). Excess iron can lead to iron-dependent cell death, or ferroptosis. DFO is a chelator, used to treat iron overload, that binds to iron and is excreted through feces or urine, thereby removing iron from the body. Specifically, DFO, consisting of three hydroxamic acid chains terminated by free amino acid groups, is able to bind ferric iron in a 1:1 stoichiometry [29]. In addition to the accumulation of excess iron, the accumulation of lipid hydroperoxides (LOOH) is another prominent feature of ferroptosis. Stockwell et al. [18] found that Fer-1 inhibits LOOH accumulation by high-throughput screening of small-molecule libraries using cellular assays. Because of this, Fer-1 has been identified as a potent inhibitor of ferroptosis.

However, there are still two bottlenecks that restrict the delivery of these ferroptosis inhibitors to treat IPF. Firstly, the molecular structure of ferroptosis inhibitors often contains aromatic cycles, which leads to poor drug solubility and low cellular uptake. Moreover, conventional oral or injectable administration of ferroptosis inhibitors confronts the problems of focal accumulation and poor selectivity in lung area, due to the first-pass effect and off-target effects. Therefore, designing a drug-delivery system with high solubilization and targeting capacity is of great significance for the ferroptosis inhibition-based treatment of IPF.

In recent years, the advancement of nanotechnology has opened up new avenues for the field of drug delivery. Various nanoplatforms have been reported to improve drug solubility via solubilization or accommodation effects, such as micelles [30], liposomes [31], nanocrystals [32], etc., and they are employed in multiple non-classical delivery routes. Formulation of ferroptosis inhibitors into nanomedicines provides an effective solution to address the bottleneck of poor drug solubility. In addition, as a prospective non-classic delivery route, pulmonary administration can effectively solve the drawbacks of oral or injectable administration, i.e., low targetability. 

Pulmonary drug delivery system (PDDS) [33] refers to the direct delivery of drugs to the lung through an inhalation device, and the lung is used as a depot to treat both local or systemic diseases. PDDS allows the delivery of therapeutic agents in the form of aerosols, powders, and droplets through the respiratory tract to the lungs using a specific inhalation-delivery device. As a non-invasive drug-delivery system, it has a distinct lung-targeting capability that can rapidly increase the concentration of drugs deposited in the lung. PDDS has unique advantages over oral and injectable routes of administration when treating respiratory diseases. Compared to oral routes of administration, PDDS can reduce the dose administered, shorten the onset of action, bypass the first-pass effect in the liver, and reduce the degradation of the drug, ultimately increasing its therapeutic effect. Compared to injectable administration, PDDS limits the entry of inhaled drugs into the circulation, reduces systemic toxicity due to poor selectivity, and meanwhile relieves patient pain and improves patient compliance [33,34,35]. Since the lesion site of IPF locates in the lung, pulmonary drug delivery is the most direct and effective approach of administration. 

Taken together, engineering ferroptosis inhibitors as inhalable nanomedicines may provide a promising therapeutic regimen for IPF, and further considerations should be taken to promote such inhalable nanomedicines from bench to bedside.

## 4. Feasible Methods to Fabricate Inhalable Nanomedicines

Currently, there is a multitude of inhalable nanomedicines to load ferroptosis inhibitors. Considering the industrialization applicability, micelles, liposomes, and nanocrystals are the most promising nanomedicines, as relevant products have witnessed commercialization [such as Cequa^®^ (cyclosporine ophthalmic solution), Arikayce^®^ (amikacin liposome inhalation suspension), and Theodur^®^ (theophylline sustained-release tablets)]. The following content will briefly summarize the major preparation methods of these nanomedicines.

### 4.1. Micelles and Preparation Method Thereof

**Figure 3:**
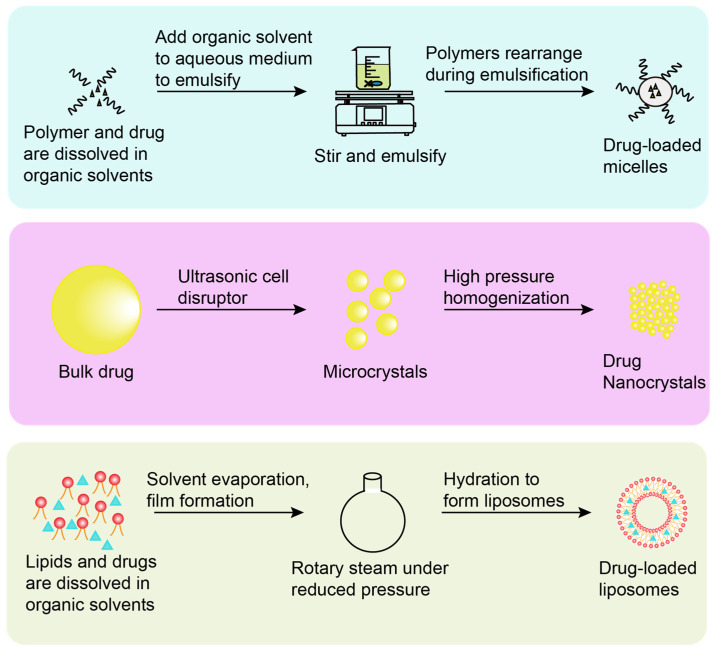
Major preparation methods of ferroptosis inhibitors laden inhalable nanomedicines.

Micelles refers to surfactant molecules in an aqueous solution that self-assemble to form an orderly arrangement of thermodynamically-stable colloidal aggregates, when the surfactant reaches a certain concentration. The micelles that are mainly studied at present are formed by amphiphilic polymer materials, and thus they are also called polymeric micelles. Polymeric micelles have the advantages of small particle size, stable structure, and strong solubilization ability. The current methods for preparing polymeric micelles include solvent-evaporation method, solid-dispersion method, dialysis method, and so on. Solvent evaporation is a widely-used approach to prepare micelles, which typically includes emulsification-solvent evaporation and self-assembly-solvent evaporation. Of note, compared with the solid-dispersion technology, micelles prepared by solvent evaporation have better stability. Compared with the dialysis method, micelles prepared via solvent evaporation have a narrower particle-size distribution, fewer side reactions, and easier operation [36]. The emulsification-solvent-evaporation method refers to dissolving the drug and the polymer in an organic solvent immiscible with water, and then adding the organic solvent to the aqueous medium for mixing and emulsification to form an oil-in-water (O/W) emulsion. The polymer rearranges to form micelles during emulsification, and the organic solvent is finally removed (Figure 3). Kim et al. [37] used an O/W emulsion-solvent evaporation method to load the poorly-soluble drug resveratrol into the core of cholesterol-conjugated polyamidoamine (PAM-Chol) micelles. The micelles incorporating the hydrophobic drug resveratrol and the therapeutic gene heme oxygenase-1 (HO-1) were inhaled to enable efficient drug delivery to the lung for the treatment of acute lung injury. Gao et al. [38] used solvent evaporation to Bellidifolin (BEL) polyethylene glycol 15-hydroxy stearate (Kolliphor HS15) loaded micelles for treating myocardial fibrosis. Compared with the free drugs, the micelles could protect the encapsulated drugs from degradation in vitro and prolong systemic drug circulation to achieve steady therapeutic effect.

In summary, we believe that the best method to prepare micelles is solvent evaporation. This method not only has a simple process, but also prepares nanomicelles with narrower particle-size distribution and better stability.

### 4.2. Nanocrystals and Preparation Method Thereof

Nanodrug crystals are stable nano-colloidal dispersion systems formed by drugs with the help of a small amount of stabilizer (surfactant or polymer material), suspending nano-scale drug molecules in a dispersion medium. The usual methods for preparing nanocrystals are “bottom-up” and “top-down” techniques, also known as self-assembly techniques and comminution techniques. The “bottom-up” technology focuses on the process of drug precipitation and nanocrystal formation, including precipitation method, supercritical fluid technology, emulsification method, and microemulsion method. Compared with the “top-down” technology, the “bottom-up” technology has the advantages of simple operation and low cost. The nanocrystals prepared by “bottom-up” technology have poor physical stability, which affects their bioavailability. At the same time, the residue of the organic solvent used in the preparation process also seriously affects the safety of the preparation. Therefore, there are no products of this technology yet. “Top-down” technology can be utilized for producing nanocrystals [39] by reducing large drug particles to nano-sized particles through mechanical force, which mainly includes media milling, high-pressure homogenization, spray drying, etc. Generally, in the preparation process of nanocrystals, it is difficult for a single preparation process to achieve the requirements of uniform particle size and good stability. Therefore, multiple methods should often be used in tandem to prepare nanocrystals. The “top-down” technique is a widely used and mature technique for the preparation of drug nanocrystals. Among them, the nanocrystals produced via the high-pressure homogenization technique have small particle sizes plus a narrow and uniform particle-size distribution (Figure 3). Currently, most of the marketed nanocrystals are oral formulations, including Rapamune^®^ (sirolimus oral solution), Emend^®^ (arepitant capsules), and Tricor^®^ (fenofibrate tablets). Nanocrystals may also be used for respiratory drug delivery, which has been widely reported in the literature. Ming et al. [40] prepared inhalable nanocrystal-embedded particles (NEPs), loaded with the Chinese herbal medicine breviscapine (BVC) and poorly-soluble compounds, using high-pressure homogenization combined with spray drying. Nanocrystal suspensions were first prepared via high-pressure homogenization, and then converted into dry particles using a laboratory-scale spray dryer. The strategy solved the problem of extremely low bioavailability of poorly water-soluble drugs. CN is a poorly-soluble drug with strong anticancer, antioxidant, and anti-inflammatory properties, and its availability in vivo is very low. Huang et al. [41] designed an inhalable mucus-penetrating nanocrystalline particle for efficient CN utilization. They prepared D-tocopheryl acid polyethylene glycol 1000 succinate (TPGS)-modified CN nanocrystals (CN-NS@TPGS) by high-pressure homogenization, which can easily escape the adsorption of mucin glycoproteins, enhancing the deposition and absorption of the drug in the lung, thereby increasing the bioavailability of CN. Experimental data demonstrated that the residence time in the lung, plasma concentration, and systemic absorption of CN after pulmonary administration can be improved by CN-NS@TPGS.

To sum up, from our point of view, the best way to prepare nanocrystals is the “top-down” technique. The “top-down” technique has a simple process and strong applicability, and the particle size of the drug nanocrystal product prepared is controllable. Besides, it has good reproducibility, and is easy to scale up production.

### 4.3. Liposomes and Preparation Method Thereof

Liposomes are a type of ultrafine spherical particle with a bilayer structure formed by self-aggregation of phospholipids as a wall material in water. Due to the amphiphilic nature of the lipids in liposomes, they can be used as candidate carriers for delivering drugs. Methods for preparing liposomes include thin-film hydration, mechanical stirring, reverse-phase evaporation, solvent injection, etc. Thin-film hydration is a widely used method for preparing liposomes (Figure 3). It refers to dissolving lipids and drugs in an organic solvent, forming a film by rotary evaporation under reduced pressure, redispersing the film in an aqueous solution at the glass transition temperature, and finally forming liposomes due to hydration. Compared with the drug leakage and degradation caused by the mechanical stirring method, thin-film hydration can avoid these two shortcomings and prepare liposomes with low permeability and high stability. Compared with the reverse-phase evaporation method, thin-film hydration has a simple preparation process and is easy to operate. Compared with the solvent-injection method, liposomes prepared via thin-film hydration have higher encapsulation efficiency [42]. Zhang et al. [43] prepared CN-loaded liposomes for inhalation to treat primary lung cancer using a thin-film hydration method. The liposomes significantly enhanced the therapeutic efficacy of CN by increasing the solubility and endocytosis. Honmane et al. [44] prepared salbutamol sulfate (SS) liposomes via thin-film hydration for the treatment of asthma. SS was the first-line drug used to treat asthma, while it could be eliminated by first-pass metabolism in the liver after oral administration. Besides, the oral administration of high-dose SS could produce adverse effects including dyspnea, palpitations, and anxiety. The pulmonary administration of SS liposomes overcame these side effects and achieved highly effective asthma treatment.

From the above discussion, we believe that the thin-film hydration method is the best solution for the preparation of liposomes. The thin-film hydration method is of simple operation. In addition, liposomes prepared by the thin-film hydration method have high stability, which allows safe and leak-free drug delivery to target organs.

### 4.4. Liposomes Are the Most Promising Nanomedicines

Herein, we presented three promising nanomedicines and the advantages and challenges of different preparation methods. The authors argued that liposomes might be the most promising and robust nanomedicines for carrying ferroptosis inhibitors. The reasons are as follows.

In extensive studies of PDDS, it has been found that the vast majority of drug monomers are metabolized and cleared within four hours after direct PDDS administration, thus requiring frequent multiple doses to maintain efficacy [44]. It was documented that loading the drug into a delivery vehicle is a good way to solve the problem of rapid drug monomer clearance, and liposomes, especially the hydrophilic modified liposomes, can better solve the problem of rapid drug clearance as a delivery vehicle.

**Figure 4:**
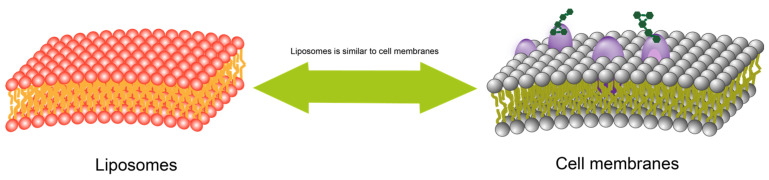
Comparison of liposome structure and biomolecular membrane structure.

Compared to micelles, liposomes possess better physicochemical stability. Therefore, liposomes can be protected from environmental factors and protected from enzymatic degradation before reaching the lesion site, which can improve drug stability, reduce drug toxicity, and exert better therapeutic effects. Additionally, the components (mainly phospholipids and cholesterol) and structure (lipid bilayer) of liposomes are similar to cell membranes, which makes them highly biocompatible and biodegradable (Figure 4). Compared with drug nanocrystals, liposomes can be structurally modified to be tissue-targeted, prolonging the effective retention time of liposomes at the lesion site and even enabling efficient targeted drug delivery. With a structure similar to that of biological membranes, modification on the membrane surface allows liposomes to acquire specific biological properties that better mimic the structure and function of cells in vivo to perform a range of complex tasks [44]. From this angle, liposomes may even be regarded as “nanocells”.

Numerous studies have been performed to achieve better lung targeting by modifying liposomes to target surface molecules to specific cells in the lung. Yan et al. [45] developed a lung-targeting cationic liposome formulation to encapsulate anti-miR-21, in which the cationic liposome is positively charged, which not only provides a positive charge for the liposome to bind the nucleic acid, but also enhances the interaction between the liposome and the negatively charged cell membrane, thereby improving cellular uptake efficiency and enabling lung targeting. Pandolfi et al. [46] used hyaluronic acid-modified liposomes as an innovative targeting delivery system for pulmonary fibrosis cells to achieve better therapeutic results by targeting CD44, a specific receptor highly expressed on pulmonary fibrosis cells, allowing more drugs to be engulfed by pulmonary fibrosis cells. Yang et al. [47] constructed pathological collagen-targeted and penetrating liposomes (DP-CC) for the delivery of dual antifibrotic drugs. Among them, the liposomes were co-modified by collagen-binding peptide (CBP) and collagenase (COL). Due to the high affinity of CBP for collagen, which could help the carrier to recognize pathological collagen and target fibrotic lung effectively, higher drug accumulation at the site of the lesion was achieved. With further in-depth research on IPF targets and biomarkers, we believe that more sophisticated liposome modification strategies for IPF targeted therapy can be developed soon.

It was worth mentioning that there are already-marketed inhalable liposomes such as the abovementioned Arikayce^®^ (amikacin liposome inhalation suspension). It is an inhalable liposome nanosuspension for the treatment of non-tuberculous mycobacterial (NTM) lung disease caused by *Mycobacterium avium* and was launched in 2018 [48]. This product delivers amikacin directly to the lung, where the drug is taken up by NTM-infected lung macrophages, prolonging amikacin release in the lung while reducing systemic toxicity by reducing systemic exposure. The commercial product mirrors the industrial and clinical translation potential of liposomes for inhalation.

As mentioned above, thin-film hydration is the most suitable preparation method for liposomes in our opinion, and hence we suggest that researchers in the field adapt this method for inhaled liposome development.

## 5. Proper Devices for Inhalable Liposomes Delivery

**Figure 5:**
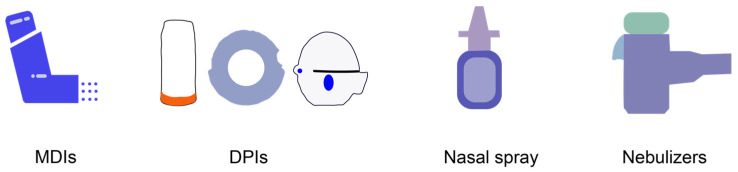
Schematic diagram of commonly used PDDS devices.

The next consideration is bench-to-bedside translation. To render inhalable liposomes applicable in clinical application, a PDDS device should be employed. Currently, the most commonly used PDDSs in clinical practice are metered-dose inhalers (MDIs), dry powder inhalers (DPIs), nasal spray, and nebulizers [49,50,51,52] (Figure 5). Hence, we may choose to transform liposomes into MDIs, DPIs, nasal spray and nebulizers.

MDIs mean that the drug and the propellants (e.g., HFA 134a) are enclosed together in a pressurized container with a dosing valve, and are sprayed out by means of an external force, which have the advantage of being stable, cheap, and simple to install [53]. However, MDIs require a high level of patient cooperation (inhalation while the drug is being injected) yet at least 50% of patients are poorly cooperative, making drug delivery inefficient. In addition, the addition of the propellants poses a challenge to the physicochemical stability of the liposomes.

DPIs refer to a preparation in which the dry powder of the drug itself or the dry powder of the drug mixed with a carrier (e.g., lactose), stored in a capsule or a multi-dose reservoir, is inhaled actively by the patient using a special device [54]. Compared with MDIs, DPIs eliminate the difficulty of poor coordination of patients, but are limited by the hygroscopicity of drugs, high requirements for device design, and notable product differences. In addition, if the liposomes are prepared as DPIs, the liposomes need to be dehydrated, where the structure of the liposomes may be destroyed to affect the stability. 

Nasal sprays refer to nasal liquid formulations made from a clear solution, suspension, or emulsion of raw drug and suitable excipients (e.g., glycol) for atomization [55]. Compared to MDIs and DPIs, the nasal-inhalation route lacks precision in dosing and may involve the nasal-brain pathway, where the liposomes may enter the brain and cause unpredictable side effects.

Nebulizers are preparations in which a drug-containing solution is nebulized into small droplets by a nebulizer for inhalation [56]. Compared to MDIs, DPIs and nasal spray, nebulizers are simple to prescribe, less irritating, and unaffected by the patient’s breathing behavior. Most importantly, liposomes can readily remain stable in a nebulizer formulation. Therefore, nebulizers are more suitable for clinical applications of inhalable liposomes. Related marketed products for this dosage form include amikacin inhalation suspension (Arikayce^®^) and aminotransol inhalation solution (Cayston^®^).

To conclude, nebulizers are the optimal PDDS device to deliver liposomes loaded with ferroptosis inhibitors for the treatment of IPF, bringing benefits to IPF patients. We recommend that relevant investigators pay more attention to this system.

## 6. Conclusions and Outlook

In summary, we propose that inhibiting ferroptosis could slow down the pathogenesis of IPF, and that formulating a ferroptosis inhibitor as an inhaled nanomedicine holds great promise for the treatment of IPF. In addition, we also described a series of classical nanoengineering technologies to construct the inhalable nanosized ferroptosis-inhibiting systems, such as micelles, liposomes, and nanocrystal preparations.

Although engineering ferroptosis inhibitors as inhalable nanomedicines for the highly efficient treatment of IPF is promising, there are some concerns about the safety of ferroptosis inhibitors and inhalable nanomedicines. First of all, ferroptosis is a recently discovered type of cellular death that generally occurs in various types of cells, that is, ferroptosis can occur in both normal cells and IPF-related cells. In the case of IPF, inhibition of ferroptosis in diseased alveolar cells slowed the progression of IPF, but the consequences of inhibiting ferroptosis in normal cells could not be assessed. Therefore, the safety issues posed by the delivery of ferroptosis inhibitors still need to be further explored. Secondly, drug delivery systems such as micelles, nanocrystals, and liposomes raise some important issues. For example, after the micelles enter the human body, their stability is greatly weakened, which leads to the premature release of the drug and the loss of targeting effect, while the effective drug content has not yet accumulated in the lesion. In addition, premature drug-release will have toxic side effects on normal cells. For nanocrystals, there is a general problem of Ostwald ripening in the nanocrystal systems, which causes crystal growth of the product and formation of large-scale crystals, affecting the particle size and its distribution. At the same time, the residual organic solvents can bring serious toxic side effects. As to liposomes, it is not yet clear whether the membrane-fusion phenomenon occurs upon contact with IPF-related and normal cells provokes an unpredictable outcome. How to solve these problems will also be the subject of our long-term exploration. 

In the future, we will experimentally verify the therapeutic effect of inhalable ferroptosis inhibitor-loaded nanomedicines on IPF. With the in-depth study of ferroptosis and inhaled nanomedicines, especially the industrialization and translation aspects, it is foreseeable that inhaled ferroptosis inhibitors may provide alternative treatment options for IPF. 

## Data Availability

The data presented in this study are available on reauest from the corresponding author.
